# Evaluation of *Haloferax mediterranei* Strain R4 Capabilities for Cadmium Removal from Brines

**DOI:** 10.3390/md21020072

**Published:** 2023-01-21

**Authors:** Iraide Saez-Zamacona, Guillermo Grindlay, Rosa María Martínez-Espinosa

**Affiliations:** 1 Multidisciplinary Institute for Environmental Studies “Ramón Margalef”, University of Alicante, Ap. 99, E-03080 Alicante, Spain; 2 Department of Analytical Chemistry, Nutrition and Food Sciences, Faculty of Sciences, University of Alicante, Ap. 99, E-03080 Alicante, Spain; 3 Biochemistry, Molecular Biology, Edaphology and Agricultural Chemistry Department, Faculty of Sciences, University of Alicante, Ap. 99, E-03080 Alicante, Spain

**Keywords:** cadmium, bioremediation, haloarchaea, metals, brine

## Abstract

*Haloferax mediterranei* has revealed a high bioremediation potential for several inorganic anions (e.g., nitrates and nitrites) and metals from hypersaline waters and brines. However, it is unclear, to date, whether this microorganism allows Cd (II) bioremediation. Consequently, the main objective of this work was to assess the Cd (II) bioremediation potential of *Hfx. mediterranei* R4. To this end, *Hfx. mediterranei* cell growth rate and metal bioaccumulation were investigated using different culture media (complex, CM, and defined medium, DM) containing Cd (II) up to 1 mM. In addition, the elemental profile of the biomass (i.e., Al, Ba, Ca, Co, Cu, Fe, K, Mg, Mn, Na, Ni, Sr and Zn) has also been monitored to gain insight into the metabolic processes that may be taking place at the intracellular level for Cd (II) removal. Because of the formation of CdS precipitate, CM is not a suitable culture media for evaluating Cd bioremediation since metal concentration could not be appropriately controlled. When operating in DM, it was observed that the cell doubling time increases three times in the presence of Cd (II). *Hfx. mediterranei* can bioaccumulate Cd, showing the highest significant accumulation at concentrations of 0.4 mM (108 ± 12 mg Cd/g dry tissue). Finally, the presence of Cd (II) affects the content of K, Mg, Mn and Zn in the biomass, by increasing K levels up to 27 ± 18% and Mn up to 310 ± 140% and reducing Mg levels up to 55 ± 36% and Zn up to 30 ± 4%. These results suggest that different mechanisms are involved in Cd (II) tolerance by *Hfx. mediterranei*, resulting in increasing the cell concentration of stress-tolerant elements in the biomass (K and Mn), while lowering the concentration of elements which Cd (II) competes with (Mg and Zn), and that all affects the physiological response of the organism by decreasing its growth rate.

## 1. Introduction

In recent years, large areas of the Earth have been affected by industrialization processes that have triggered an increase in pollution of the natural environment [1,2,3]. Metal pollution caused by natural or anthropogenic processes [4] tends to persist in the environment indefinitely [5,6,7] due to its low ability to be removed, thus becoming more hazardous to the environment [6]. Although some metals are essential for the proper functioning of organisms, others do not have these functions and are, therefore, considered non-essential, such as Cd [8]. This element has been defined as a “cumulative poison” [9] due to its highly toxic potential, making it among the priority pollutants to be controlled by various environmental monitoring programs [10]. According to the World Health Organization [11], Cd can cause great damage to human health, mainly affecting the kidneys [12], altering Ca (II) metabolism [13], weakening bones, causing pneumonia and pulmonary edema after inhalation, and favoring the development of kidney and prostate cancer [14], among other pathologies. Thus, it is necessary to find a solution for the removal of this pollutant from the environment.

Among the different methods by which metals can be removed from the environment, bioremediation has become the most sought-after technique as it is considered the safest, cleanest, most economical, and environmentally friendly process [3,8]. Because organisms can adopt different strategies to tolerate these pollutants, the bioremediation technique offers the possibility of eliminating pollutants using the natural biological activity of certain organisms, which, unlike traditional techniques, makes it a low-cost technique with low technological difficulty, high social acceptance and with the capacity to be carried out at the site where the pollution has occurred [7,15]. Most bioremediation research has been focused on organisms of the Bacteria domain [3], with a special focus on organic pollutants. Nevertheless, in recent years, the molecular biology of extremophilic archaea has aroused great interest in the field of bioremediation [16,17,18,19]. Within haloarchaea (“salt-loving archaea”), *Haloferax mediterranei* is a Gram (-), aerobic, moderately halophilic (compared to some species of the genera *Halobacterium* and *Halococcus*) and neutral media haloarchaea that was isolated for the first time in Santa Pola’s salt ponds, Alicante, Spain [20,21]. Since its discovery, *Hfx. mediterranei* has been used as: (i) a model organism in molecular biology studies [22,23]; (ii) a cell factory for carotenoid and biopolymer production [24,25]; and (iii) a bioremediation microorganism for inorganic anions removal (e.g., NO_2_^−^, NO_3_^−^, ClO_3_^−^_,_ ClO_4_^−^, and BrO_3_^−^) from hypersaline wastewater generated from the chemical, pharmaceutical, agricultural and aquaculture industries as well as from desalination water plants [16,18,19,21,26,27,28].

To date, the use of *Haloferax mediterranei* for metal bioremediation has not been extensively investigated [19,29,30] (Table 1). For instance, Popescu and Dumitru in 2009 [31] tested the bioremediation capacity of *Hfx. mediterranei* in the presence of Pb (II), Cr (VI), Zn (II) and Ni (II), obtaining metal reductions close to 100%. Recently, Matarredona and co-workers [18] showed the resistance and accumulation capacity of this organism to the metals As (V), Co (II), Li (I) and Ni (II), as well as their accumulation inside the cells at concentrations of 20 nM, 1.2 mM, >500 mM and 1.6 mM, respectively. Though Cd tolerance by *Haloferax* strain BBK2 (*Hfx. volcanii*) was investigated [32], no information has been specifically reported for *Hfx. mediterranei*, particularly in liquid media mimicking brines and hypersaline wastewater samples. To the best of our knowledge, there is just a single work conducted by Nieto and co-workers evaluating this matter, but it was focused on cell cultures in agar plates in order to get MIC values [30]. Therefore, this work aimed to evaluate the bioremediation capacity of *Hfx. mediterranei* strain R4 for hypersaline waters containing Cd (II). To this end, *Hfx. mediterranei* cell growth rate and metal bioaccumulation were investigated using different culture media (complex, CM, and defined medium, DM) containing Cd up to 1 mM. To gain insight into the metabolic processes that may be taking place at the intracellular level for Cd removal, the elemental profile of the biomass (i.e., Al, Ba, Ca, Co, Cu, Fe, K, Mg, Mn, Na, Ni, Sr and Zn) has been monitored under Cd exposure. Consequently, this work contributes to understanding the effect of Cd (II) on haloarchaea metabolism and the potential uses of haloarchaea as cell factories for the bioremediation of metals.

## 2. Results

### 2.1. Influence of the Incubation Medium on Cell Growth Rate: CM vs. DM

*Hfx. mediterranei* was grown in two different media, complex medium (CM) and defined medium (DM) (see Section 4 for chemical composition), since it was expected that the number of nutrients available in the media may affect the tolerance of the organisms being exposed to some stressors, making them more tolerant when they do not have to compete for the resources [33].

Cells grew in CM for all the concentrations of Cd (II) tested (i.e., 0–1.0 mM). The higher the concentration of Cd (II) in the medium, the slower the cell growth found. Moreover, it was observed that the maximum values of optical density for Cd-containing treatments were lower than in the control. Interestingly, all the media showed a yellow-colored precipitate that could be easily distinguished from the pink pellet of the cells (see Figure S1B of the Supplementary Materials). The precipitate formation was also noticed before inoculating all media with cells, thus ruling out that the precipitate was not a product of them (see Figure S1A of the Supplementary Materials). In fact, the precipitate formation was only dependent on Cd (II) concentration. The higher the Cd (II) concentration, the higher the amount of precipitate formed. The precipitate composition was specifically determined by different chemical analytical methodologies (e.g., qualitative assays for anions/cations identification and ICP-OES), being formed by CdS [34]. Because Cd levels cannot be appropriately controlled for CM, this medium was discarded for further studies.

**Figure 1:**
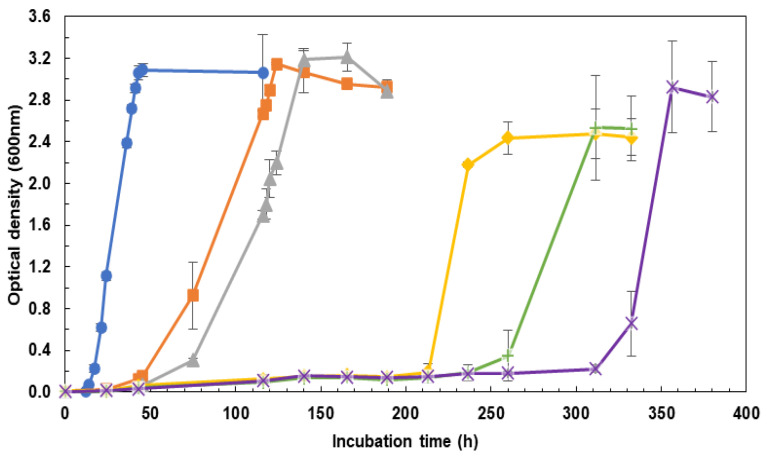
Growth curves of *Hfx. mediterranei* grown in the presence of Cd (II) in DM. Note: (●) 0 mM Cd (II) (control), (■) 0.2 mM Cd (II), (▲) 0.4 mM Cd (II), (◆) 0.6 mM Cd (II), (**+**) 0.8 mM Cd (II) and (**×**) 1.0 mM Cd (II).

When operating with DM, no CdS precipitate is formed for all the Cd (II) levels tested, thus allowing to evaluate *Hfx. mediterranei* cell growth rate in a controlled manner. As previously noticed for CM, the lag phase was enhanced when increasing Cd (II) concentration levels (Figure 1). Significant differences were found in doubling times (F_5,12_ = 326.1; *p*-value < 0.01) between the control (6.170 ± 0.010) and the treatments (16.6 ± 1.8), in which treatments exposed to Cd (II) increased their doubling rate in almost three times compared to the control. No differences were noticed, however, among treatments (0 mM Cd > 0.2 mM Cd = 0.4 mM Cd = 0.6 mM Cd = 0.8 mM Cd = 1 mM Cd from the results of Tukey’s HSD test). Unlike the experiments with CM, no significant reduction in the growth for any of the Cd (II) concentrations tested was observed (F_5,12_ = 2.269; *p*-value = 0.114) (OD = 2.8 ± 0.3). According to these findings, *Hfx. mediterranei* is able to grow even at concentrations of 1 mM of Cd (II) (Figure 1).

### 2.2. Cd Accumulation by Haloferax mediterranei in DM and Assessment of Bioremediation Capacity

**Figure 2:**
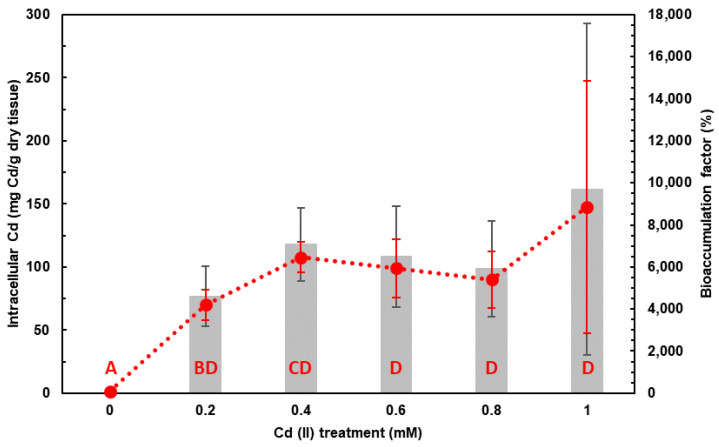
Cd accumulation by *Hfx. mediterranei* cells grown in the presence of Cd (II) in DM. Note: (··●··) Intracellular Cd accumulation and (■) Cd bioconcentration factor (BF); different letters indicate statistically significant differences among treatments from Tukey’s HSD test for intracellular Cd concentration; letters are assigned in alphabetical order from the lowest value to the highest.

The elemental analysis revealed that *Hfx. mediterranei* can accumulate Cd inside ranging from 70 (0.2 mM) to 150 (1 mM) mg/g dry tissue. Significant differences in the intracellular Cd concentrations (F_5,12_ = 3.764, *p*-value < 0.05) were found between the control and all treatments. However, only treatments 0.2 and 0.4 mM showed significant differences within treatments, accumulating up to 70 ± 12 mg Cd/g dry tissue when exposed to 0.2 mM Cd (II) and 108 ± 12 mg Cd/g dry tissue when exposed to 0.4 mM Cd (II) (Figure 2).

The bioaccumulation factor of Cd in *Hfx. mediterranei* did not show any significant difference among treatments (F_4,10_ = 0.679, *p*-value = 0.622), with a slight tendency between treatments 0.2 mM of Cd (II) (4600 ± 1400%) and 0.4 mM of Cd (II) (7100 ± 1800%) (F_1,4_ = 3.58, *p*-value = 0.131) when analyzing these two treatments independently. However, it can be observed how it has the same behavior as the intracellular Cd concentration increasing the variability at higher Cd (II) concentrations (Figure 2).

### 2.3. Influence of Cd in Cell Elemental Profile in DM

To gain insight into *Hfx. mediterranei* tolerance to Cd (II), changes in elemental profile have been examined since some elements play a crucial role in metabolic processes in charge of detoxification. To this end, 13 elements (Al, Ba, Ca, Co, Cu, Fe, K, Mg, Mn, Na, Ni, Sr and Zn) have been investigated. This study was carried out with treatments up to 0.4 mM due to the high variability of data shown by the experiments in which higher concentrations of Cd (II) were used.

**Figure 3:**
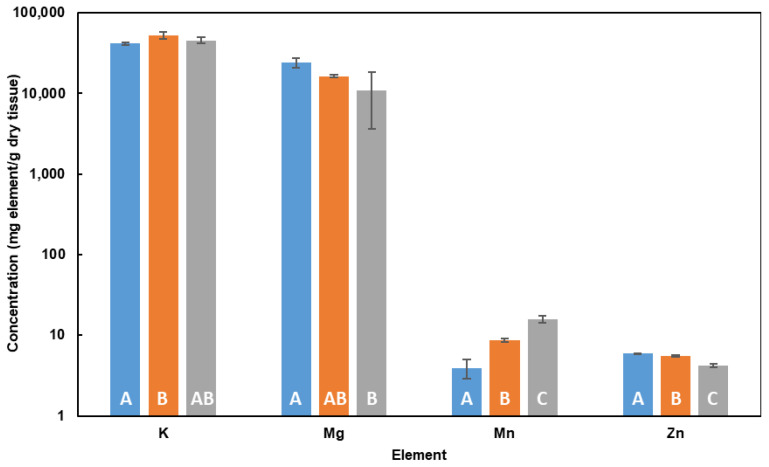
Variation of the concentration of the elemental profile in *Hfx. mediterranei* cells grown in the presence of Cd (II) in DM for elements K, Mg, Mn and Zn. Note: (■) 0 mM Cd (II) (control), (■) 0.2 mM Cd (II), (■) 0.4 mM Cd (II); concentration axis is represented in log scale; different letters indicate statistically significant differences among treatments from Tukey’s HSD test.

Among the elements investigated, only K, Mg, Mn and Zn intracellular concentration levels were statistically affected by Cd (II) treatments (Table S3). The intracellular concentration of K and Mn increased (F_2,6_ = 5.802, *p*-value < 0.05 and F_2,6_ = 98.73, *p*-value < 0.001, respectively). K concentration increased 27 ± 18% when the cells were exposed to 0.2 mM of Cd (II), with a non-significant slight decrease at higher concentrations, indicating that the presence of Cd (II) seems to trigger the concentration of K, independently of its concentration. On the contrary, intracellular Mn increased significantly not only compared to the control, but also among the treatments, increasing its concentrations compared to control in 120 ± 70% for 0.2 mM Cd (II) treatment, and 310 ± 140% for 0.4 mM Cd (II) treatment. As regards Mg and Zn, concentration levels decrease (F_2,6_ = 6.035, *p*-value < 0.05 and F_2,6_ = 114.3, *p*-value < 0.001, respectively) in the presence of Cd (II) in the medium (Figure 3). The intracellular concentration of Mg decreases in the presence of higher Cd (II) concentrations in the medium as a significant decrease of 55 ± 36% is observed in the treatment 0.4 mM and not for the 0.2 mM one. However, in the case of Zn, its intracellular concentration decreases significantly when increasing Cd (II) concentration, decreasing 6 ± 3% in the presence of 0.2 mM Cd (II), and 30 ± 4% in the presence of 0.4 mM Cd (II).

## 3. Discussion

### 3.1. Influence of the Incubation Medium on Cell Growth Rate: CM vs. DM

The appearance of the CdS precipitate in the CM for Cd (II) treatments suggests that this culture medium might not be appropriate for Cd bioremediation experiments due to a lack of control of the metal concentration to which the organism is being exposed to. Vido and co-workers [35] reported that Cd (II) affects the redox balance of thiol groups present in yeast amino acids meaning that it is possible that the thiol groups of the amino acids in the yeast extract used in the experiment are promoting Cd precipitates in the form of CdS. Furthermore, Lagorce and co-workers [36] showed that even trying to minimize the presence of inorganic ligands containing cysteine (an amino acid with a thiol group), Cd (II) reacts with the thiol groups and precipitates in the form of CdS. According to these data, special care is required when studying elements prone to react with thiol groups since they could lead to the metal-sulfide precipitate formation. DM is, a priori, a more suitable medium for Cd (II) bioremediation experiments due to the absence of CdS precipitate, thus allowing control of the concentration the organism is exposed to. Moreover, DM media mimics better brine composition, osmosis concentrates and industrial wastewaters, making this medium more representative of the natural environment and potential wastewater to be bioremediated [37].

The increase in the lag phase at higher Cd (II) concentrations in the medium and the increase in the doubling time in the presence of Cd (II) result in a slower growth rate compared to the control. These results agree with the observation of Das and co-workers [32] working with *Hfx. volcanii* in brines. These authors found that the growth of the haloarchaeon slowed down for Cd (II) concentration levels up to 4 mM. According to this data, it is expected that *Hfx. mediterranei* could also grow under Cd (II) concentration levels above 1 mM, but no specific experiment was carried out to estimate the minimum inhibitory concentration (MIC). Even so, *Hfx. mediterranei* demonstrated to be able to grow in the presence of 1 mM of Cd (II) (i.e., 112.4 mg L^−1^), a representative value of highly Cd (II) contaminated wastewaters, as the threshold for Cd in waters is set at 1.5 μg L^−1^ [33].

In the present study, treatments containing Cd (II) increased their doubling time three times that of the control, meaning that the population would take three times more time than the control to double the population size. Similar results were observed in Matarredona and co-workers [18] when *Hxf. mediterranei* was exposed to the lowest levels of Ni (II) (0.5 mM) as the doubling time also increased three times compared to the control. Nevertheless, the lowest concentration of Co (II) (0.2 mM) that they tested resulted in a higher increase (6.6 times that of the control), demonstrating that metal ions similar to Cd (II) can affect the cells in different ways as Ni (II) showed similar results to Cd (II), increasing the doubling rate three times, whereas Co (II) seems to affect the cells as at lower concentrations—the doubling time increases.

### 3.2. Cd Accumulation by Haloferax mediterranei in DM

Our results show that Cd concentration in the cell increases significantly up to the treatment of 0.4 mM of Cd (II), showing the maximum Cd accumulation at that concentration (108 ± 12 mg Cd/g dry tissue, 7100 ± 1800% with respect to control). Similar findings were reported by Das and co-workers [32] with *Hfx. volcanii* in which the maximum accumulation of Cd occurred at 0.5 mM of Cd (II) (21.08%), but showing lower levels of accumulation compared to that of *Hfx. mediterranei* shown in the present work, suggesting that *Hfx. mediterranei* is a more suitable candidate for Cd bioremediation than *Hfx. volcanii*. A priori, it can be expected that a higher Cd (II) exposure would give rise to higher intracellular levels of this element. However, within experimental uncertainties in this work, this phenomenon has not been observed. These results suggest that osmoregulation processes could significantly influence Cd accumulation by *Hfx. mediterranei.* Nevertheless, Popescu and Dumitru [31] in their study reported that when *Hfx. mediterranei* was exposed to some metals similar to Cd (II) (i.e., Pb (II)) the production of anionic nature exopolysaccharides increased as a metal tolerance response, so it cannot be discarded that metals might also be retained in the EPS. Regarding the strategy to store Cd inside the cell, no specific mechanism has been studied in the present work. However, Das and co-workers [32] described that *Hfx. volcanii* accumulated Cd intracellularly as CdS nanoparticles (CdSNPs). The formation of CdS NPs has its basis in the metal chelation that happens in the cytoplasm where S of cysteine-rich peptides [38] may react with intracellular Cd to form CdSNPs. Thus, it can be hypothesized that, as *Hfx. mediterranei* and *Hfx. volcanii* belong to the same genus and show similar behavior, *Hfx. mediterranei* may show similar results as *Hfx. volcanii*. Nevertheless, there is still the need for research to verify this hypothesis, and future studies will address this idea as it has already been observed in some preliminary results that *Hfx. mediterranei* is able to form NPs when exposed to other elements [39].

Finally, and as a general discussion, the high variability of data at higher Cd (II) concentrations can be explained by the inoculum. Although it is intended that all cells of the inoculum are in the same phase of growth, when using high volumes of inoculum there is a higher probability of having cells in different phases of growth. Therefore, the Cd may not affect them in the same way, and higher concentrations of Cd (II) may trigger different tolerance strategies. Consequently, they may not tolerate and accumulate similarly. For this reason, in future studies, smaller inoculum volumes should be used to try to ensure that the cells at the pre-inoculum are in the same phase of growth showing a similar metabolic stage. The volume of the inoculum here used is that previously added to the cultures in most of the studies reported from *Haloferax*, which is an optimal inoculum to monitor accurately cellular growth, oxygen availability and feasibility of the bioremediation process to be designed based on the results obtained in batch cultures. The main problem with the use of lower volumes of inoculum resides in the fact that the incubation times of the cultures to reach the stationary phase of growth are higher compared to those observed in this analysis, which prevents a precise assessment of the efficiency of the bioremediation process.to Several studies using preadapted inoculum could also be conducted in the future to shed light on this variability.

### 3.3. Influence of Cd in Cell Elemental Profile in DM

The presence of Cd (II) in the medium significantly affects the concentration of several elements (K, Mg, Mn and Zn) involved in different metabolic processes (i.e., osmotic regulation and stress tolerance). K plays a crucial role in the activation of several intracellular enzymes, osmoregulation, and regulation of internal pH [40]. *Hfx. mediterranei* may be accumulating higher concentrations of K so that it can fulfil its function by activating enzymes responsible for transforming this metal into its less toxic form or by activating enzymes that can enhance the desorption of this pollutant. Furthermore, it has been reported that K, specifically KCl, is among the essential intracellular salts in halophilic organisms [41], so the intracellular accumulation of this element is related to osmoregulation processes. Similarly, the decrease in Mg in the presence of Cd (II) may be linked to osmoregulation processes. As more Cd (II) ions are entering the cell, the organism needs to keep the ionic balance and may pull out there to get it. However, no data have been found regarding this hypothesis, so more research should be conducted to assess this behavior better. Nevertheless, considering that many enzymes in haloarchaea are Mg dependent, the decrease in Mg in the presence of Cd (II) could negatively affect the stability and activity of several halophilic enzymes and chaperones, thus contributing to limitations in terms of metabolic activity and cellular growth [42,43,44].

The decrease in intracellular Zn as the concentration of Cd (II) in the medium increases may be due to the competition between Cd (II) and Zn (II) [8]. Zn is an essential micronutrient that plays an important role in numerous physiological processes, as it is a cofactor for the six major functional classes of enzymes [45] and is particularly important in the maintenance of protein structure [8,46,47]. The main mechanisms that maintain stable cellular Zn (II) concentrations are limited to highly regulated processes of Zn (II) import, metal ion sequestration by metallochaperones and Zn (II) export across the cytoplasmic membrane. Zn (II) import and export are carried out by a mixture of unique bacterial transport proteins which have evolved to enable the flow of metal ions from the same group across the cytoplasmic membrane, meaning that similar ions (with similar electronic structure and chemical similarity), such as Cd (II), may use these mechanisms to pass through the membrane [48]. Moreover, once inside the cell, Cd (II) can interact with Zn (II)-dependent enzymes, such as carboxypeptidases, causing the cells to efflux Zn (II) outwards as Cd (II) takes its place [48].

To explain the higher intracellular Mn concentration with Cd (II), it should be considered that this element has antioxidant activity. Culotta and Daly [49] described Mn complexes as antioxidant agents, playing a role similar to that of glutathione in eukaryotic cells, conferring a protective barrier to the cell in the presence of Cd (II) in the environment. Glutathione, for instance, an antioxidant agent, has been reported to be involved in numerous cellular processes including protecting cells from toxic compounds being among the first lines of defense against Cd (II) toxicity [50]. Thus, it can be expected that Mn-dependent mechanisms in this haloarchaeon, as the mechanisms of the glutathione in eukaryotic cells, are those conferring stress tolerance to redox processes to the microorganism. On the other hand, as mentioned for Mg, several halophilic enzymes have been described as Mn dependent [42,43,44].

## 4. Materials and Methods

### 4.1. Reagents

For all the dilutions prepared, high-purity water (resistivity > 18 MΩ) from a Direct-Q 3V water purification system (Millipore Inc., Paris, France) was used.

For the incubation of *Hfx. mediterranei*, saltwater was prepared using CaCl_2_·H_2_O (Labken, Barcelona, Spain), KCl, NaBr, NaCl and NaHCO_3_ (Panreac, Castellar del Valles, Spain), and MgCl_2_·6H_2_O and MgSO_4_·7H_2_O (VWR Chemicals, Radnor, PA, USA). Two different incubation media were prepared from the saltwater: CM by adding yeast extract (Condalab, Madrid, Spain) and DM by adding NH_4_Cl, D(±)-Anhydrous glucose, Na_2_HPO_4_, NaH_2_PO_4_ (Panreac, Castellar del Valles, Spain), and Tris(hydroxymethyl)-aminomethane and FeCl_3_ from (Merck, Darmstadt, Germany). Cd (II) stock solution was prepared using the salt CdCl_2_·2.5 H_2_O (Merck, Darmstadt, Germany), and the pH of the medium was adjusted using HCl (Merck, Darmstadt, Germany) and NaOH (Panreac, Castellar del Valles, Spain).

Finally, for ICP analysis, sample digestion was conducted using 69% HNO_3_ (Panreac, Castellar del Valles, Spain) and the calibration standard solutions were prepared from the ICP-IV multi-elemental standard as well as Mo, Sc and Ru monoelemental standards, all reagents and purchased from Merck (Darmstadt, Germany).

### 4.2. Cd Resistance and Growth Kinetics

The effect of Cd (II) in the growth of the haloarchaea was assessed in two different media, CM and DM, in the presence of Cd (II) in the concentration range from 0 to 0.4 mM for CM, and from 0 to 1 mM, at intervals of 0.2, in both cases. The concentration of Cd (II) was added from a 0.1 M Cd (II) stock solution prepared from the salt CdCl_2_·2.5H_2_O.

*Hfx. mediterranei* strain R4 (ATCC33500) was grown to stationary phase in CM and DM with saltwater prepared at 30% according to the composition described by Rodríguez-Valera and co-workers [20], which contained (per liter): 234.0 g NaCl, 59.3 g MgSO_4_·7H_2_O, 41.5 g MgCl_2_·6H_2_O, 6.0 g KCl, 2.9 CaCl_2_·2H_2_O, 0.7 g NaBr and 0.2 g NaHCO_3_. From this solution, two different media were prepared with a final salt concentration of 20%. Apart from the salt content, CM contained 5 mg mL^−1^ of yeast extract and the corresponding Cd (II) concentration for each treatment, and DM consisted of 50 mM Tris(hydroxymethyl)-aminomethane, 0.015 M NH_4_Cl, the corresponding concentration of Cd (II), 0.5 M of inorganic phosphate (Na_2_HPO_4_-NaH_2_PO_4_ buffered at pH = 7.5), 0.5% glucose and 5 mg mL^−1^ of FeCl_3_. Finally, all culture media were adjusted to pH 7.2–7.3 by adding HCl and NaOH before autoclaving (autoclave conditions: 121 °C, 20 min at 1–2 atm). The last three compounds of the DM were added after autoclaving the culture media as they are thermolabile compounds.

After the complete preparation of the culture media, they were inoculated at 0.2% of the medium volume by adding 100 µL of pre-inoculum grown in CM without Cd (II) to inoculate CM treatments, and with pre-inoculum grown in DM without Cd to inoculate DM treatments. In both cases, pre-inoculums were allowed to grow to the stationary phase (OD = 2.8 ± 0.3) under optimal conditions without Cd (II).

*Hfx. mediterranei* was grown in an aerobic medium in 0.5 L Erlenmeyer flasks filled with culture medium at 10% of the total volume (50 mL) in an incubator at 42 °C and 170 rpm (optimal conditions) to facilitate the aeration of the cultures and improve oxygenation. The growth of the cultures was monitored by periodic optical density measurements using a Cary 60 UV–Vis spectrophotometer (Agilent, Santa Clara, CA, USA) at a wavelength of 600 nm. The periodicity of the measurements was determined by the growth rate of each of the cultures.

### 4.3. Sample Treatment for Elemental Analysis

Once the cultures reached the stationary phase, they were centrifuged at 720 rpm for 20 min at a temperature of 4 °C to try to preserve the integrity of the samples. After centrifugation, the pellet was washed twice with salt water at 15%, which consisted of resuspending the pellet in salt water at 15%, centrifuging under the same conditions as previously described, and removing the supernatant. After, samples were dried in an oven until the complete elimination of moisture to obtain the dry mass. Then, they were digested by the addition of 1.5 g 69% HNO_3_ in a sand bath at a temperature of approximately 70 °C for 6 h for complete digestion of the samples. If lower temperatures than indicated are used, the digestion is not completely carried out, thus affecting the accuracy and precision of the results. Next, digests were made up to 20 g for a final HNO_3_ concentration of 5% *w*/*w*. Finally, aliquots of the latter solution were diluted 1:10 and 1:100. Nitric acid concentration was kept constant for these solutions at 5% *w*/*w*.

According to *Hfx. mediterranei* biomass composition, 1:100 and undigested samples were, respectively, employed for the analysis of major (Ca, K, Mg and Na) and trace elements (Fe, Mn, and Zn) by ICP-OES. To improve accuracy and minimize matrix effects, prior to ICP-OES determinations, 1:100 and undiluted samples digests were spiked with Sc as the internal standard (final concentration 0.5 mg L). Determination of ultra-trace elements by ICP-MS was carried out using 1:10 and undiluted samples. The former solution was used for measuring Al, Ba, Cu, Ni and Sr, while the latter allowed Co determinations. Both sample solutions were spiked with Ru as the internal standard (final concentration of 10 µg L^−1^) [51,52].

### 4.4. Elemental Analysis

Elemental analysis of *Hfx. mediterranei* biomass composition was performed by ICP-AES (720 ICP-AES, Agilent, Santa Clara, CA, USA) and ICP-MS (8900x ICP-MS, Agilent, Santa Clara, CA, USA). Cadmium, major and trace elemental determination was performed by ICP-OES. Determination of ultra-trace elements was, however, carried out by ICP-MS due to its higher sensitivity and lower limits of detection regarding ICP-OES [53]. Details of the operating conditions employed for both techniques are gathered in Table S2 (see Supplementary Materials). Elemental analysis by ICP-based techniques was based on external calibration using matrix-matched standards (nitric acid 5%) and internal standardization to correct for matrix effects and signal drift [54,55]. As has already been mentioned, Sc and Ru were, respectively, used as internal standards for ICP-OES and ICP-MS determinations [51,52].

### 4.5. Data Statistical Analysis

The R statistical software [54] was used for data analysis. Most analyses were performed based on a one-factor experimental design using the concentration of Cd (II) in the culture medium as a fixed and orthogonal factor with 6 levels (0 (control), 0.2, 0.4, 0.6, 0.8, and 1.0 mM Cd (II)).

In order to observe the possible effect of Cd (II) on the growth of *Hfx. mediterranei*, analysis of variance (ANOVA) was performed for the variables, maximum optical density and growth rate, in the exponential phase of the growth curve, where this last parameter was obtained from the slope of the curve and, after, the doubling time was calculated as in Matarredona and co-workers [18].

To assess if *Hfx. mediterranei* was able to accumulate Cd, ANOVA was performed for intracellular Cd concentration among treatments. Moreover, to evaluate the bioremediation potential of this organism, ANOVA of the bioaccumulation factor (BF) was carried out analyzing the BF among treatments. The BF was calculated as follows Equation (1):

BF (%) = (Cd concentration in treatment cells/Cd concentration in control cells) × 100(1)

Finally, ANOVA was also carried out for each of the elements measured to determine the variations in the elemental profile of *Hfx. mediterranei* in relation to the concentration of Cd (II) in the culture medium. In this case, the experimental design only contained 3 levels (0 (control), 0.2 and 0.4 mM of Cd (II)) due to the high variability of data noticed for Cd treatments higher than 0.4 mM.

Prior to ANOVA, the homogeneity of variance was checked with Cochran’s C-test [55]. When ANOVA showed significant differences, a posteriori test was performed using Tukey’s HSD test [56].

In order to perform the statistical analysis, those elements that presented a concentration lower than the detection limit of the measuring instrumentation were assigned a concentration value equal to the detection limit of each corresponding element, since the fact that the actual concentration could not be determined is due to the detection limit of the equipment and not because the element is not present. In addition, statistical analysis was not performed for culture media grown on CM due to interference from a Cd precipitate described in the results. Finally, for the elemental analysis, only data for elements that showed consistency in their behavior were analyzed.

## 5. Conclusions

Results in this work support the use of *Hfx. mediterranei* strain R4 in the bioremediation of hypersaline waters containing Cd (II) up to 1 mM. When exposed to Cd (II), this haloarchaea accumulates Cd inside the cell, thus decreasing the cell growth rate. Moreover, it has been observed that Cd (II) affects the elemental profile of *Hfx. mediterranei* by increasing the expression of elements related to stress tolerance (K and Mn), specifically oxidative stress tolerance, and decreasing the presence of some ions it competes with or in order to maintain the ionic balance (Mg and Zn).

As an overview of all the mechanisms proposed in the previous sections, and according to the results obtained in the present study, we can suggest two ways in which Cd is being stored in the cell: (i) attached to the EPS membrane of anionic nature, or (ii) inside the cell, probably as CdSNPs as it was found in Das and co-workers [32] with the similar haloarchaea *Hfx. volcanii*. It is clear that the presence of Cd (II) overexpresses elements related to stress tolerance (osmotic imbalance and redox processes), suggesting the activation of the corresponding metabolic mechanisms to deal with the stress caused by Cd (II). At the same time, when Cd (II) enters the cells, it competes with Zn, which is essential for the proper functioning of the physiology of the cells, as well as the structure of some proteins. The activation of all these metabolic mechanisms, as well as the decrease in elements essential for proper physiological functioning and the energy the organism must invest in it, affects physiological responses by increasing the time the organisms grow. However, it should be highlighted that the two proposed mechanisms of Cd storage are not incompatible, as a fraction of Cd might be retained in the EPS, whereas other fractions might enter the cell.

Finally, the present work sheds light on a range of possibilities for further studies related to the bioremediation of metals by *Hfx. mediterranei*, particularly Cd, and makes clear the need for future studies in this line to fulfill the gaps not solved yet, as with the characterization of the molecular machinery involved in Cd removal (by in silico approaches and/or *in lab* experiments), the description of the proteome of the cells grown in the presence of Cd or the upscaling of the process here described for the removal of Cd in order to calculate accurately the efficiency of this bioremediation approach.

## Figures and Tables

**Table 1:** Research conducted with *Haloferax mediterranei* and metals. Note: NA = not applicable.

Pollutant	Tolerated Concentration	Description	Reference
As (V)	20 mM	Toxicity tests carried out by agar dilution plate method in which MIC values were obtained for each metal analyzed; within all haloarchaea tested *Hfx. mediterranei* showed the highest tolerance to Cd (II).	[30]
Ag (I)	0.5 mM		
Ni (II)	2.5 mM		
Co (II)	1 mM		
Pb (II)	20 mM		
Cd (II)	2.5 mM		
Cr (VI)	5 mM		
Hg (II)	0.01 mM		
Zn (II)	0.5 mM		
Cu (II)	1 mM		
Cr (VI)	5 mM	Both tolerance and accumulation of these metals were observed, as well as a reduction close to 100%; The organisms removed the metals more efficiently when glucose was added at a concentration of 2%; in the presence of Pb (II) and glucose at 2% the organisms were able to synthesize a greater amount of exomucopolysaccharides.	[31]
Pb (II)	2.5 mM		
Ni (II)	2.5 mM		
Zn (II)	1 mM		
As (V)	20 mM	The higher the concentration of the metal in the medium, the higher the concentration of the metal in the cells.	[18]
Co (II)	1.2 mM		
Li (I)	>500 mM		
Ni (II)	1.6 mM		
Cu (II)	NA	Through bioinformatic tools, genes coding for Cu import and export, coding for proteins involved in antioxidant mechanisms and coding for genes involved in Cu metabolism were identified, showing the potential of this organism to bioremediate Cu.	[19]

## Supplementary Materials

The following supporting information can be downloaded at: https://www.mdpi.com/article/10.3390/md21020072/s1, Figure S1: Appearance of the yellow precipitate CdS in CM in (A) non-centrifuged CM without cells and (B) centrifuged CM with cells. Note: pink pellet represents the cell biomass. Table S1: Microorganisms with bioremediation potential for different metals. Note: NS = non stated. Table S2: ICP-OES and ICP-MS operating conditions. Table S3: Summary of ANOVA results for elemental variation analysis according to the different Cd treatments (0, 0.2 and 0.4 mM of Cd (II)).
