# Supplementary material for: Evaluation of Haloferax mediterranei Strain R4 Capabilities for Cadmium Removal from Brines
Source: Mar Drugs. 2023 Jan 21;21(2):72. doi: 10.3390/md21020072 (PMC9960891; doi:10.3390/md21020072)
Supplement: Supplementary file 1 [file marinedrugs-21-00072-s001.zip › marinedrugs-2132908-supplementary.pdf]

Supplementary Material

Evaluation of *Haloferax mediterranei* Strain R4 Capabilities for Cadmium Removal from Brines

Iraide Saez-Zamacona [1], Guillermo Grindlay [2] and Rosa María Martínez-Espinosa [1,3,*]

[1] Multidisciplinary Institute for Environmental Studies "Ramón Margalef", University of Alicante, Ap. 99, E-03080 Alicante, Spain
[2] Department of Analytical Chemistry, Nutrition and Food Sciences, Faculty of Sciences, University of Alicante, Ap. 99, E-03080 Alicante, Spain
[3] Biochemistry, Molecular Biology, Edaphology and Agricultural Chemistry Department, Faculty of Sciences, University of Alicante, Ap. 99, E-03080 Alicante, Spain
* Correspondence: rosa.martinez@ua.es; Tel.: +349-6590-3400 (ext. 1258 or 8841)

Table S1. Microorganisms with bioremediation potential for different metals. Note: NS=Non stated.

Specie	Metal	Concentration	Field	Description	Reference
Chlamydomonas sp.	Cd (II)	< 0.8 mM	Organisms isolated from Cd-contaminated sites; *Ex situ* laboratory assay	Tolerance and accumulation of Cd; The higher Cd concentration and the larger exposure time result in a higher accumulation of Cd	[1]
Thiobacillus ferroxidans DSM 583	Cd (II)	1 M	*Ex situ* laboratory under way of being implemented in the treatment of real industrial discharges	Growth decreases when [Cd] ≥ 50mM; It accumulates Cd at acidic pH (1.6-6)	[2]
Halococcus salifodinae BK6	Cd (II)	2 mM	*Ex situ* laboratory assay	Gradual reduction of growth and pigmentation when Cd concentration increases	[3]
Haloferax volcanii BBK2		1 mM		Growth reduces when [Cd] ≥ 0.5mM	
Haloarcula japonica BS2		2 mM		Gradual reduction of growth and pigmentation when Cd concentration increases	
Halorubrum sp. BS17		< 0.5 mM		There is not tolerance to Cd at tested concentrations	
Haloferax volcanii BBK2	Cd (II)	1 mM	*Ex situ* laboratory assay	Tolerance to 4mM of Cd showing a significant growth reduction; The maximum accumulation of Cd was at [Cd] = 0.5mM	[4]
Pseudomonas stutzeri LA3	Cu (II)	400 mg L^{-1}	*Ex situ* laboratory assay	Removal percentage is indirectly proportional to Cu (II) concentration in the medium; Highest removal percentage was at [Cu (II)] = 50 mg L^{-1}	[5]
Thermococcus gammatolerans Ej3	Cd (II)<br>Co (II)<br>Zn (II)<br>Ni (II)<br>Cu (II)<br>As (II)	2 mM<br>2 mM<br>2 mM<br>0.5 mM<br>0.5 mM<br>0.25 mM	*Ex situ* laboratory assay	Cd tolerance is caused by the coding of about a hundred genes that are expressed 120 minutes after Cd exposure	[6]
Methanosarcina acetovirans C2A	Cd (II)	100 µM	*Ex situ* laboratory assay	Cd induces methanogenesis	[7]

Sulfolobus metalicus DSM 6482	Cd (II)	5 mM	*Ex situ* laboratory assay	ATPases efflux heavy metals outwards the cell to detoxify the intracellular environment	[8]
	Cu (II)	>200 mM			
Sulfolobus solfataricus	Cd (II)	< 0.05 mM			
	Cu (II)	NS			

Table S2. ICP-OES and ICP-MS operating conditions.

	ICP-OES	ICP-MS
Plasma forward power (W)	1400	1550
	Argon flow rate (L min^{-1})	
Plasma	15	15
Auxiliary	1.50	0.90
Nebulizer	0.70	1.09
Sample uptake rate (mL min^{-1})	0.5	0.3
View mode	Axial	-
Cell gas		He
Cell gas flow (mL min^{-1})		4
Number of replicates	3	3
Scanning mode	-	Peak Jump
Points per Peak	-	3
Dwell time (μs)	-	150
Number of sweeps	-	40
Element (wavelength, nm) / Nuclide	Ca (422.673); Cd (226.502); Fe (238.204); K (404.721); Mg (285.213); Mn (257.610); Na (588.995); Zn (213.857)	$^{27}Al^{\pm}$; $^{59}Co^{\pm}$; $^{63}Cu^{2\pm}$ $^{60}Ni^{\pm}$; $^{88}Sr^{\pm}$; $^{137}Ba^{\pm}$
Internal standard	Sc (391.182, 361.383)	$^{101}Ru^{\pm}$

Table S3. Summary of ANOVA results for elemental variation analysis according to the different Cd treatments (0, 0.2 and 0.4 mM of Cd (II)).

Source of variance	F-value ($F_{2,6}$)	p-value	Tukey HSD test
Al	0.002	n.s.	-
Ba	1.529	n.s.	-
Ca	30.51	n.s.	-
Co	0.647	n.s.	-
Cu	1.146	n.s.	-
Fe	1.465	n.s.	-
K	5.802	< 0.05	0 < 0.2; 0.2 = 0.4; 0 = 0.4
Mg	6.035	< 0.05	0 = 0.2; 0.2 = 0.4; 0 > 0.4
Mn	98.730	< 0.001	0 < 0.2 < 0.4
Na	4.473	n.s.	-
Ni	1.885	n.s.	-
Sr	3.709	n.s.	-
Zn	114.300	< 0.001	0 > 0.2 > 0.4

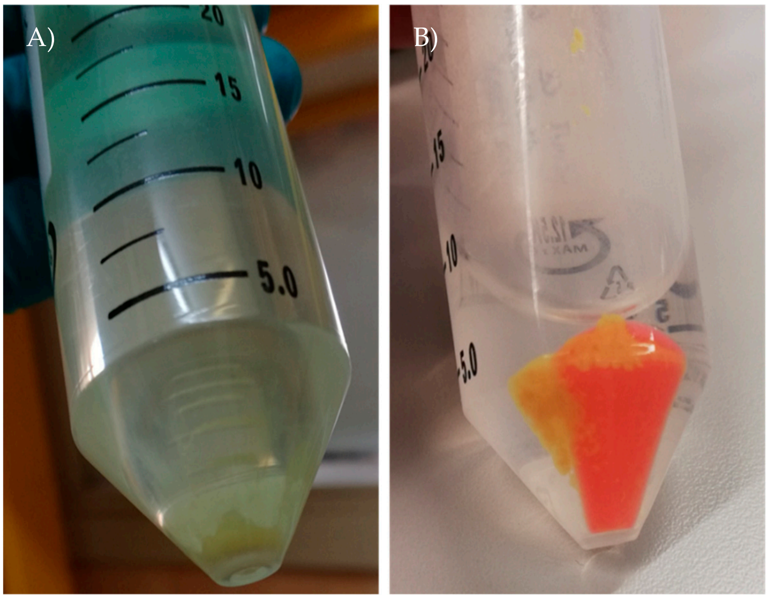

Figure S1. Appearance of the yellow precipitate CdS in CM. **A**) No centrifuged CM without cells and **B**) centrifuged CM with cells. Note: pink pellet corresponds to the cell biomass.

References

1. Aguilera, A.; Amils, R. Tolerance to cadmium in *Chlamydomonas* sp. (Chlorophyta) strains isolated from an extreme acidic environment, the Tinto River (SW, Spain). *Aquat. Toxicol.* **2005**, *75*, 316–329. https://doi.org/10.1016/j.aquatox.2005.09.00.
2. Baillet, F.; Magnin, J.P.; Cheruy, A.; Ozil, P. Cadmium Tolerance and Uptake by a *Thiobacillus ferrooxidans* Biomass. *Environ. Technol.* **1997**, *18*, 631–638. https://doi.org/10.1080/09593331808616581.
3. Chaudhary, A.; Pasha, M.I.; Salgaonkar, B.B.; Braganca, J.M. Cadmium Tolerance by Haloarchaeal Strains Isolated from Solar Salterns of Goa, India. *Int. J. Biosci. Biochem. Bioinf.* **2014**, *4*, 1–6. https://doi.org/10.7763/ijbbb.2014.v4.299.
4. Chackraborty, J.; Das, S. Characterization and cadmium-resistant gene expression of biofilm-forming marine bacterium *Pseudomonas aeruginosa* JP-11. *Environ. Sci. Pollut. Res.* **2014**, *21*, 14188–14201. https://doi.org/10.1007/s11356-014-3308-7.
5. Palanivel, T.M.; Sivakumar, N.; Al-Ansari, A.; Victor, R. Bioremediation of copper by active cells of *Pseudomonas stutzeri* LA3 isolated from an abandoned copper mine soil. *J. Environ. Manag.* **2020**, *253*, 109706. https://doi.org/10.1016/j.jenvman.2019.109706.
6. Lagorce, A.; Fourçans, A.; Dutertre, M.; Bouyssiere, B.; Zivanovic, Y.; Confalonieri, F. Genome-wide transcriptional response of the Archaeon *Thermococcus gammatolerans* to Cadmium. *PLoS ONE* **2012**, *7*, e41935. https://doi.org/10.1371/journal.pone.0041935.
7. Lira-Silva, E.; Santiago-Martínez, M.G.; Hernández-Juárez, V.; García-Contreras, R.; Moreno-Sánchez, R.; Jasso-Chávez, R. Activation of Methanogenesis by Cadmium in the Marine Archaeon *Methanosarcina acetivorans*. *PLoS ONE* **2012**, *7*, e48779. https://doi.org/10.1371/journal.pone.0048779.
8. Orell, A.; Remonsellez, F.; Arancibia, R.; Jerez, C.A. Molecular Characterization of Copper and Cadmium Resistance Determinants in the Biomining Thermoacidophilic Archaeon *Sulfolobus metallicus*. *Archaea* **2013**, *2013*, 289236. https://doi.org/10.1155/2013/289236.
